# Time Course of Immediate Early Gene Protein Expression in the Spinal Cord following Conditioning Stimulation of the Sciatic Nerve in Rats

**DOI:** 10.1371/journal.pone.0123604

**Published:** 2015-04-10

**Authors:** Ognjen Bojovic, Debabrata Panja, Margarethe Bittins, Clive R. Bramham, Arne Tjølsen

**Affiliations:** Department of Biomedicine, University of Bergen, Jonas Lies vei 91, 5009 Bergen, Norway; University of Texas at Dallas, UNITED STATES

## Abstract

Long-term potentiation induced by conditioning electrical stimulation of afferent fibers is a widely studied form of synaptic plasticity in the brain and the spinal cord. In the spinal cord dorsal horn, long-term potentiation is induced by a series of high-frequency trains applied to primary afferent fibers. Conditioning stimulation (CS) of sciatic nerve primary afferent fibers also induces expression of immediate early gene proteins in the lumbar spinal cord. However, the time course of immediate early gene expression and the rostral-caudal distribution of expression in the spinal cord have not been systematically studied. Here, we examined the effects of sciatic nerve conditioning stimulation (10 stimulus trains, 0.5 ms stimuli, 7.2 mA, 100 Hz, train duration 2 s, 8 s intervals between trains) on cellular expression of immediate early genes, Arc, c-Fos and Zif268, in anesthetized rats. Immunohistochemical analysis was performed on sagittal sections obtained from Th13- L5 segments of the spinal cord at 1, 2, 3, 6 and 12 h post-CS. Strikingly, all immediate early genes exhibited a monophasic increase in expression with peak increases detected in dorsal horn neurons at 2 hours post-CS. Regional analysis showed peak increases at the location between the L3 and L4 spinal segments. Both Arc, c-Fos and Zif268 remained significantly elevated at 2 hours, followed by a sharp decrease in immediate early gene expression between 2 and 3 hours post-CS. Colocalization analysis performed at 2 hours post-CS showed that all c-Fos and Zif268 neurons were positive for Arc, while 30% and 43% of Arc positive neurons were positive for c-Fos and Zif268, respectively. The present study identifies the spinal cord level and time course of immediate early gene (IEGP) expression of relevance for analysis of IEGPs function in neuronal plasticity and nociception.

## Introduction

Long term potentiation (LTP) is one of the widely studied phenomena of synaptic plasticity which is predominantly produced in the brain as a response to conditioning stimulation (CS) [1]. LTP and long-term depression (LTD) have been first researched in hippocampus studies [2, 3] and later described as long lasting changes in the synaptic efficiency regardless of the specific location of a chemical synapse [4]. The LTP model has been often used in spinal cord (SC) studies in terms of determining the cellular and synaptic changes in synaptic plasticity [4]. LTP has been divided in early and late phases, and it has been found that the early-phase LTP requires trafficking of pre-formed proteins while the late-phase LTP requires *de novo* protein synthesis [5]. Many studies have shown that immediate-early gene proteins Zif268 (synonyms: zinc finger protein 225; Egr-1) and activity-regulated cytoskeleton-associated protein (Arc) are required for the process of late-phase LTP establishment [5, 6]. The synthesis of Arc is needed for the phosphorylation of cofilin and the expansion of F-actin cytoskeleton during LTP consolidation [7]., Zif268 has been shown to have an important role in LTP formation [8], as well as that the development of persistent pain states are associated with immediate early gene formation [9]. The role of Zif268 remains undefined but it has been suggested that zif268 may contribute to signaling pathways involved in synaptic potentiation [10].

Further, it has been thought that CS of primary afferent fibers leads to changes in synaptic transmission in the dorsal horn that were described as a component of central nociceptive sensitization [4]. Central sensitization represents the increase of synaptic efficacy in somatosensory neurons in the dorsal horn as a consequence of strong peripheral noxious stimulation [11]. Enhancement of synaptic transmission is responsible for the reduction of pain threshold, increment of pain responses and distribution of pain sensitivity to non-stimulated areas [11]. Both firing of dorsal horn neurons and field potentials have significant relevance for changes in pain sensitivity and central sensitization [1]. Therefore we have used the term long term facilitation (LTF) for the increase of the output of action potentials from the dorsal horn neurons [1].

The process of acquiring, processing and transmitting nociceptive information in the spinal cord is performed mainly by nociceptive specific neurons (NS) and wide dynamic range neurons (WDR) [12]. Within the first two laminae of the spinal cord, the NS neurons are the most abundant neurons and respond to intense, often pain-producing stimuli [12, 13]. We have studied laminae I and II, as defined in the research of Molander et al [14]. In the deeper laminae (V and VI), WDR neurons are abundantly present and react in a graded manner to gentle touch, stronger mechanical and noxious stimulations [15]. Nociceptive information is conducted from the periphery across myelinated Aδ and unmyelinated C fibers towards the dorsal horn where they make synaptic contacts with neurons of higher order [16–18]. The most effective type of stimulation to induce LTP in the spinal cord is shown to be a series of high-frequency (e.g. 100 Hz) trains of electric stimulation [4]. Furthermore, peripheral stimulation of afferent fibers was shown to lead to an increase of expression of c-Fos mRNA in spinal cord neurons [19], and synaptic Arc protein [20].

By focusing on early time points (1 h [21]; 2 h [22]; 3 h [1, 23]) and smaller areas of the SC (L4- L5 [1, 24] L3-L5 [25], L2-L6 [23]), previous studies have resulted in conflicting and fragmented data. In addition, studies have shown that 8 h after CS, c-Fos showed a biphasic immunoreactivity pattern in deeper laminas (V-VII, X) [26]. With the fact that afferent C fibres terminate in lamina I, II and V, we aimed to determine the effects of 100 Hz sciatic nerve CS in anesthetized rats on Arc, c-Fos and Zif268 expression in the dorsal horn SC. Here we studied a wider area of the SC (TH13- L5 for Arc, L3-L5 for c-Fos and Zif268), from early (1 h) to late (12 h) time points, with the goal to determine the rostral- caudal SC level with maximal IEGP and investigate the possible biphasic IEGP immunoreactivity pattern. Since Arc is known to have a very specific subcellular distribution in hippocampal neurons [7, 27], we also addressed the cellular and subcellular expression pattern of Arc in dorsal horn neurons after sciatic CS by confocal microscopy and colocalisation analysis.

This study addresses the temporal and spatial expression of IEGPs in the SC in response to CS and is an essential groundwork for further studies regarding nociception in the lumbar SC.

## Results

### Stimulation-induced Arc expression is maximal at the midpoint between the L3 and L4 segments

Our first aim was to determine the post-stimulation time points for maximum IEGP expression in response to CS. We performed immunohistochemistry staining on SC sagittal sections for all sets of animals subjected to CS. We counted the number of positive cells for each individual IEGP. For all further experiments, the animals were divided into three sets of six animals. Each set of animals consisted of one animal from each group (sham, 1, 2, 3, 6, 12 hours).

**Fig 1:**
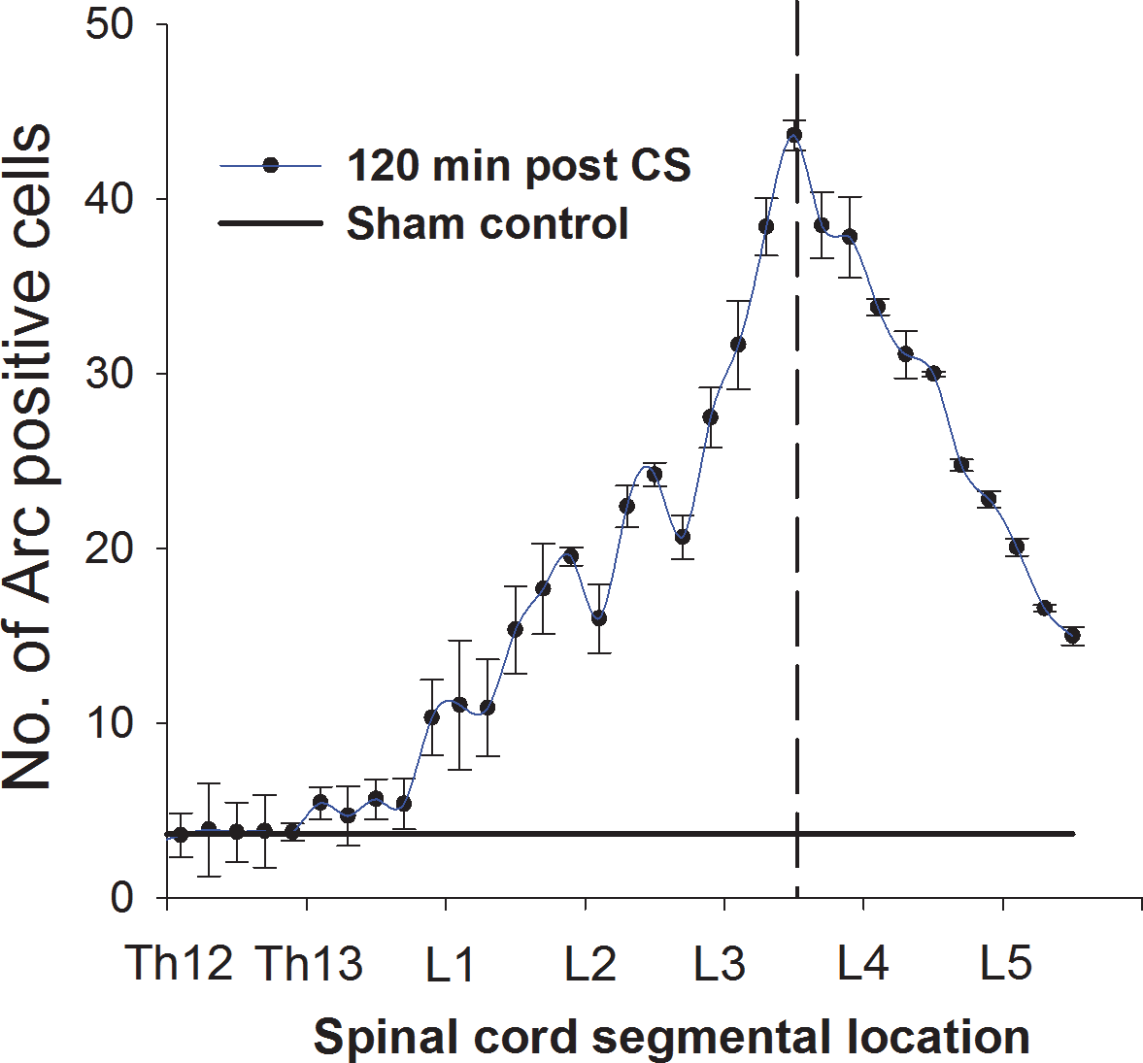
Distribution of Arc positive cells along segments of the spinal cord. Number of Arc positive cells relative to spinal cord segmental location. Vertical segmented line represents the position between the L3 and L4 segments with the highest number of Arc positive cells. Data points represent means ± SEM of four consecutive sections (400μm) from each of three rats (N = 3 animals). The control graph shows the average number of cells in control, unstimulated rats (N = 3).

For subsequent experiments it was essential to determine a smaller and focused spatial region of the SC, which was most responsive to CS. We first determined the SC rostral-caudal location of maximal CS-induced Arc expressions. We conducted a series of experiments to generate data to create a distribution curve showing the number of Arc-positive cells per cross-section as a function of its location in the SC (Fig 1). We analyzed a larger area of the SC rostral and caudal from the midpoint between the L2 and L3 segments (L2/L3 ± 7000 μm) in three rats. The midpoint location is the SC location between the L2 and L3 spinal segments referred to in this text as L2/L3. The maximal number of Arc-positive cells was found at the L3/L4 (Fig 1).

### Arc, c-Fos and Zif268 immunoreactivity peaked at 2 h post-CS

**Fig 2:**
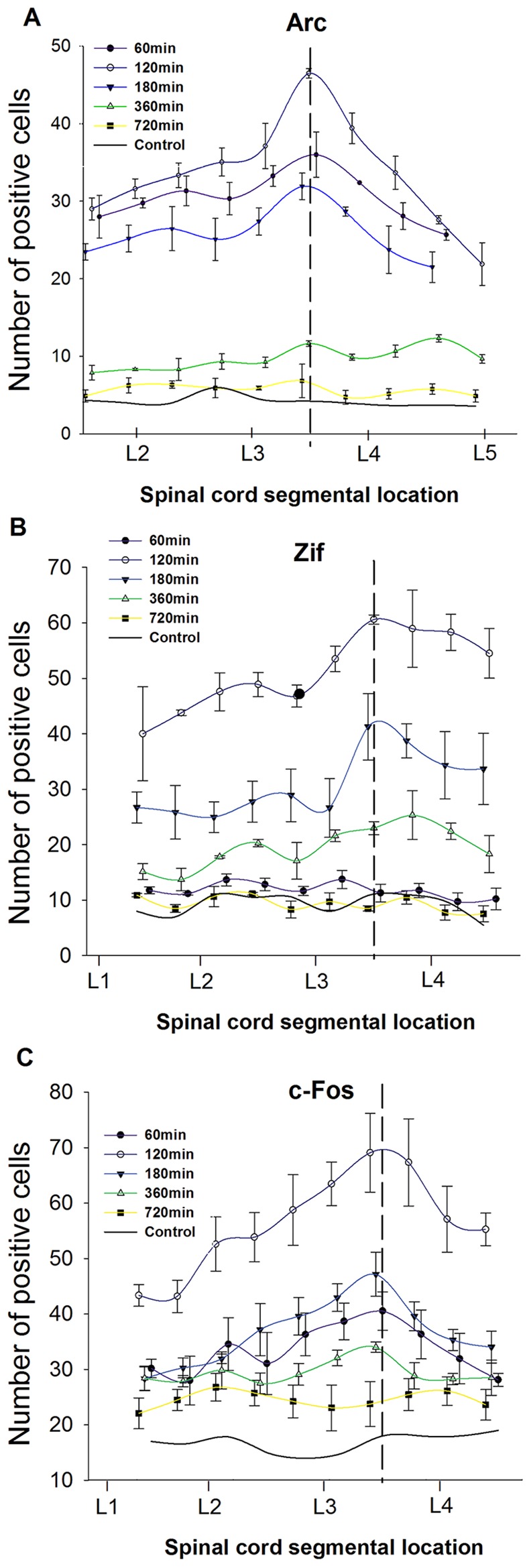
Distribution of Arc, Zif268 and c-Fos positive cells along the spinal cord 1, 2, 3, 6 and 12 h after CS. For each time point, for each IEGP the mean of four consecutive sections (400μm) from each animal (N = 3) was calculated, and the data points represent the means ± S.E.M. of these values. Control lines represent the number of positive cells in non- stimulated animals (sham operated) calculated in the same way Two-way ANOVA with Sidaks post-hoc test showed high significant/ significant effect of stimulation for all positions and time points, except: 12 h time point for all IEGPs; 2,65 mm; 3,85 mm at 6 h post CS for Arc; 0,25–1,45 mm at 6 h for c-Fos; 2,25mm, 3.05–3,85 mm at 6 h post CS for Zif268.

To study the formation and maintenance of CS-induced IEGP expression we examined Arc, c-Fos and Zif268 immunoreactivity. All animals except the sham animals were subjected to the previously described CS, and were sacrificed after different pre-defined post-stimulation periods. Simple spline scatter graphs with means (± SEM) show how the numbers of positive cells vary with time and the location in the SC (Fig 2A and 2C). The results showed that the maximum number of IEGP positive cells in all groups was found at L3/L4 2 h post-CS. The number of IEGP positive cells showed lower values both before and after the 2 h point, reaching almost the control level 12 h post-CS.

Two-way ANOVA on samples from all time points (1 h, 2 h, 3 h, 6 h and 12 h group vs. controls; P<0.005, number of cells as dependent, position from L2/L3 in mm and stimulation vs not stimulation as independent variables) showed a significant effect of stimulation (P<0.0001) and significant effect of position (P<0.0001) (Fig 2A and 2C). Sidac’s post-hoc tests showed significant effects of stimulation for all positions and time points, except: 12 h time point for all IEGPs; 2.65 mm and 3.85 mm at 6 h post CS for Arc; 0.25–1.45 mm at 6 h for c-Fos; 2.25 mm and 3.05–3.85 mm at 6 h post CS for Zif268.

In the dorsal horn on the unstimulated side, there was no significant increase of the number of IEGP positive cells. The number of positive cells on the non-stimulated side was approximately at the control level (Fig 2A and 2C).

### Immunoblot analyzis of Arc IEGP 2 h post- CS

**Fig 3:**
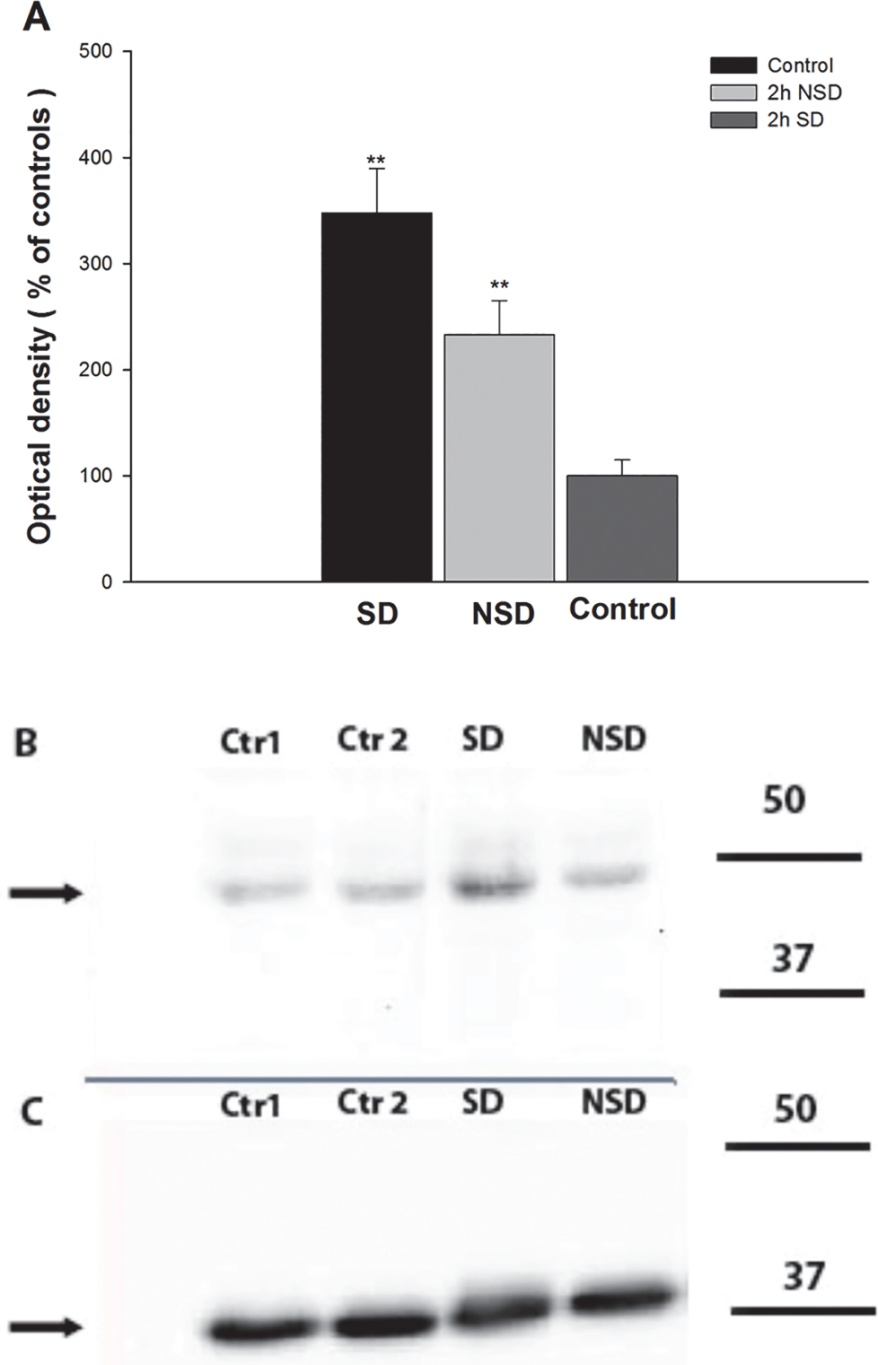
Arc protein immunoblot analysis 2 h post CS. Immunoblot analysis (Fig 3A), 2h post CS, Arc IEGP, stimulated side dorsal horn (SD), non-stimulated side dorsal horn (NSD) and control, 2mm caudal from midpoint. Data given as % of mean control values, means ± S.E.M (N = 5)(**p<0.01, unpaired Student’s T-tests vs control). Immunoblot scan (Fig 3B), for Arc protein: Ctr1,2—Sham controls; SD- Stimulated side; NSD- Non stimulated side (Production of western blot spinal cord sections described in material and methods). Immunoblot scan (Fig 3C), for GAPDH- used as a quantifiable loading control.

In order to confirm Arc expression from immunohistochemical staining procedures we conducted western blot assays using homogenates from sagittal cut micro-dissected (L3-L5) parts of SC samples. The post CS time point with the highest number of positive cells found (2 h) was used for immunoblot analysis. It was observed that after CS there was a significant increase of Arc expression on the stimulated side compared to unstimulated control SC, and a significant but lower increase of expression of Arc on the contralateral side (Fig 3A and 3C). Bar graphs show group mean (± SEM) changes based on chemoluminescence densitometry analysis. Values are expressed as a ratio between the tissue from stimulated animals and from controls. A reduction of Arc protein was found in the 3 h post CS tissue samples. This confirms that the highest expression of Arc IEGP 2 h post CS is followed by a decrease in Arc expression. Western blotting at earlier time points showed very low Arc expression on the stimulated side which was not significantly different than the level in unstimulated control SC.

### A subset of Arc labeled cells shows co-localization with Zif268 and c-Fos

**Fig 4:**
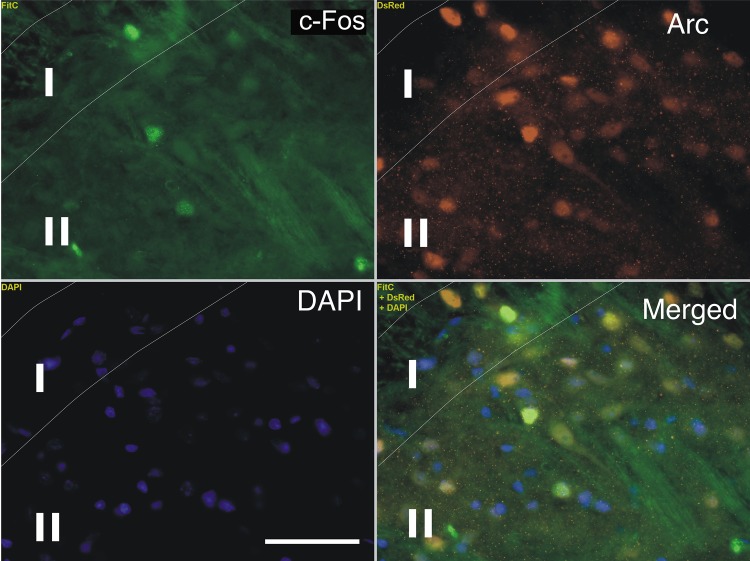
Arc and c-Fos co-localization Fluorescent micrographs. Arc was found expressed in a subpopulation of cells in the superficial dorsal horn laminae together with C-Fos. Fluorescent micrographs showing the superficial dorsal horn laminar neurons (mamina I and II, L3/L4 spinal segment)(63x; 2h post-CS) expressing Arc (red- Dsred) and C-Fos (green- FITC)(DAPI- blue- nuclear staining). Arrows indicate position of co-localization. Lamina I and II are marked with their roman numerals. Scale bar: 20 μm.

We investigated the co-localization of Arc with c-Fos or with Zif268. In order to study Arc and c-Fos or Arc and Zif268 IEGP co-localization in lamina I and II dorsal horn cells (L3/L4 spinal segment), specific primary antibodies for these IEGPs and secondary antibodies (Alexa Fluor 488 and Alexa Fluor 555) were used. We found that 30% of Arc positive neurons also were positive for c-Fos and that 43% of Arc positive cells were positive for Zif268 (N = 3; 2 h post-CS) (Fig 4) but strikingly all c-Fos and Zif268 cells were positive for Arc. Statistical analysis has previously been described.

### Arc expression remains nucleo-cytoplasmic in neurons at all time points (1 – 12 hours post CS)

**Fig 5:**
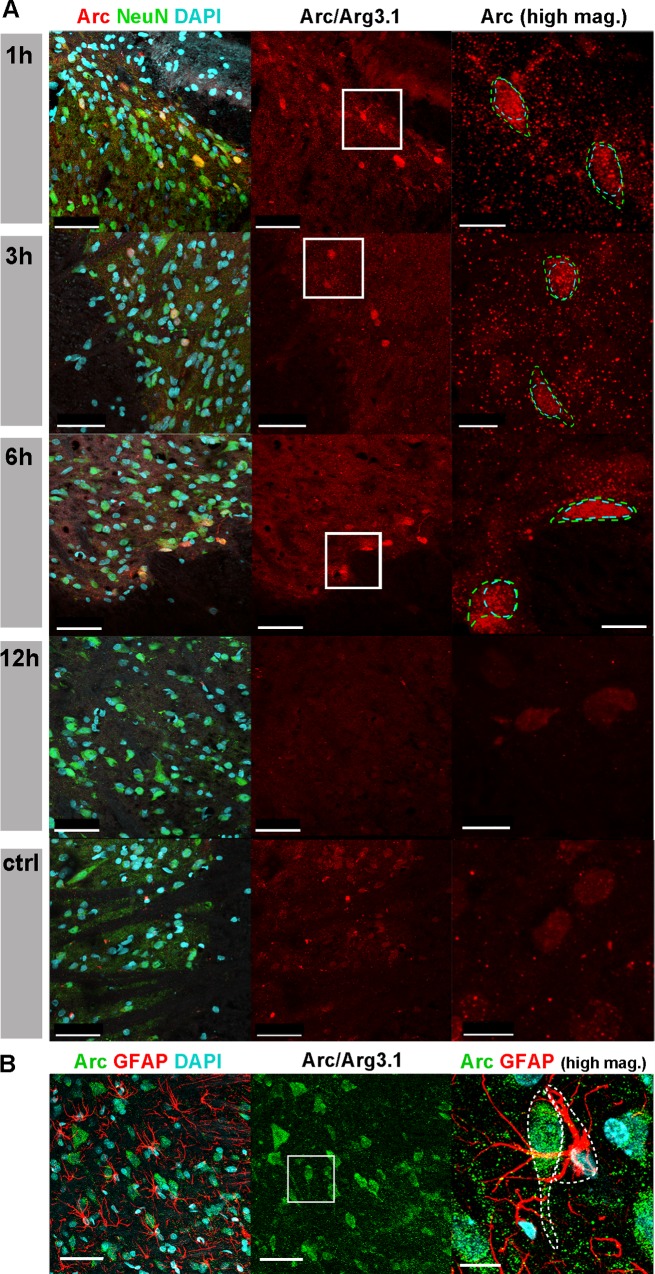
Arc protein nucleo-cytoplasmic localization. Confocal micrographs (Fig 5A) using a Leica SP5 laser scanning microscope (Leica Microsystems, Germany), with a resonant scanner, 40x oil immersion objective and 1.7x or 7-10x electronic zoom for low and high magnifications, respectively. Images are showing the superficial dorsal horn laminar cells, nucleo-cytoplasmic Arc localization from 1–12 h post CS. Arc (red- Dsred) and NeuN (green- FITC)(DAPI- blue- nuclear staining). Scale bars 50 μm and 10 μm for low and high magnifications, respectively. Co- localization of Arc and GFAP (2 h post- CS) (Fig 5B) shows no noticeable Arc staining that co- localized with glia cells. Scale bars 50 μm and 10 μm for low and high magnifications, respectively.

In order to identify the Arc-expressing cell types, we performed immunofluorescent staining using antibodies for Arc and marker proteins for neurons (NeuN) and glia cells (GFAP), respectively. Confocal fluorescence microscopy revealed that high levels of Arc were detected only in cells that also expressed NeuN, suggesting that Arc is strongly upregulated in neurons (Fig 5A). There was no noticeable co-labelling of Arc with GFAP, indicating that Arc expression is very low or absent in glia cells (Fig 5B).

Higher magnification also shows that Arc was expressed in both nuclei and cytoplasm of the neurons, and often extended into dendrites. There was no clear preference for any of these subcellular compartments, and the nucleo-cytoplasmic expression pattern remained roughly the same at all time points (Fig 5A).

## Discussion

In this study we have shown a comprehensive comparison of Arc, c-Fos and Zif268 expression in laminae I and II, 1–12 h post-CS of the sciatic nerve at the Th13- L5 (for Arc) and L3- L5 level (for c-Fos and Zif268) of the SC dorsal horn. The main findings were that (1) for all IEGPs, the maximal numbers of positive cells were observed at 2 h post CS at L3/L4 with a quantitative decrement towards earlier and later time points (1, 3, 6 and 12 h). (2) 30% of Arc positive neurons were positive for c-Fos; 43% of Arc positive neurons were positive for Zif268 (N = 3; 2 h post-CS) (Fig 4), in addition all c-Fos and Zif268 cells were positive for Arc. (3) Arc was expressed exclusively in neurons. (4) Regardless of the time post CS, the intracellular localization of Arc protein remained nucleo-cytoplasmic.

As reported in some of previous studies [1, 28] our results confirmed that the number of Arc positive cells at 2 h post-CS (the maximal point of expression) was lower than the number of c-Fos and Zif268 positive cells at the same location and at 2 h. Immunoblot analyzes showed significant Arc IEGP expression ipsilateral to stimulation 2 hours post-CS when compared to the contralateral non-stimulated side and sham operated controls. Previous studies of Zou et al [29] (c-Fos) and Dolan et al [30] (Zif268) confirmed significant Zif268 and c-Fos immunoblot expression after sciatic nerve nociceptive stimulation.

Moreover, our data suggest that IEGPs is monophasic peaking at 2 h followed by a decrement in immunoreactivity by 12 h. This is contrary to the previous report by Nyberg et al., which described a second c-Fos peak at 8 h after CS, suggesting a biphasic pattern of immunoreactivity [26]. We are unable to explain the differences between the findings in our study and those in the previously described by Nyberg et al. According to our results from confocal microscopy after immunofluorescence staining there was a higher number of Arc positive cells than cells positive for c-Fos and Zif268. Those results were contradictory to our results after DAB staining. Immunofluorescence staining and imaging may have led to a higher positive cell count for Arc since this staining contained less background staining compared to DAB staining. DAB staining due to its nature has known differences in staining between slides, may lead to higher background level and therefore masque positive cells in the background noise.

Synaptic plasticity is reported to be fundamental in the establishment of memory and pain [11]. Stimulation of nociceptive fibers by conditioning stimulation evokes facilitated transmission in dorsal horn neurons, referred to as central sensitization [11]. Central sensitization has been defined as increased synaptic efficacy formed in spinal dorsal horn somatosensory neurons following CS [11]. The increased synaptic transmission was stated to lead to the reduction of pain threshold and to amplification of responses to pain stimuli [11]. LTP has an important role in memory formation [7]. Studies on mechanisms of central sensitization and LTP have shown that there are significant similarities in synaptic plasticity ‘contributing to memory and pain’ [11].

CS induced IEGPs might play a role in the establishment of stable LTF that is involved in chronic pain development. Therefore, in order to elucidate the possible roles of SC IEGPs in the process of LTF formation and maintenance, selective IEGP block would be the future focus of our study. On the other hand, the early immunoreactivity might be a result of an activity-induced response to CS of Aδ and C afferent fibers or a result of the time limited neurotransmitter elevation in afferent nociceptive fibers. Surprisingly, in the hippocampus and striatum, c-Fos and Zif268 have shown a more rapid immunoreactivity reaching the maximum at 30 min followed by a sharp decrease [31]. In our study at all time points (1–12 h), from as early as 1h post CS, Arc was found to be present in the cellular soma and the nuclei of neurons. Similar to our results, Arc was previously found to be neuron specific [32]; in the cellular soma and nucleus [32–34]. However, contrary to our findings, Rodriguez reported Arc presence in glia cells [35].

Arc is believed to play a critical role in the process of consolidation of explicit and implicit forms of memory [7]. Nociceptive spinal cord cells expressing Arc are believed to have direct afferent input from peripheral nociceptive fibers [28]. In the hippocampus infusion of Arc antisense oligodeoxynucleotides at 2 h post- HFS led to a complete and permanent reversal of LTP (Messoudi et al., 2007). The ability of Arc antisense to reverse LTP was blocked by stabilization of F-actin, which suggested that Arc promotes LTP consolidation through regulation of actin dynamics [6]. Further, in the dentate gyrus inhibition of Arc protein production by use of intra-hippocampus infusions of Arc antisense impairs maintenance but not induction of LTP [6]. Moreover, application of broad-spectrum protein synthesis inhibitors to the spinal cord has prevented the establishment of late phase long-term facilitation (LTF) [36].

C-Fos is a proto-oncogene discovered in fibroblasts as the main gene of transformation of murine osteogenetic sarcoma virus. As a member of the Fos transcription factor family [37] c-Fos is used as a marker of general neural activity [38]. Similar to results of our study, the number of cells positive for c-Fos was previously defined as more abundant from the number of cells positive for Arc [1, 28]. Contrary to other studies [39], we did not find a second increment of c-Fos positive cells at late time points [26]. Studies revealed that peripheral CS of afferent c-fibres leads to the release of excitatory amino acids followed by NMDA receptor-dependent activation of multiple protein kinases, nitrous oxide (NO) and the expression of the immediate early gene [40] [41]. C-fos was defined as a IEGP responsible indirectly for the decrease of pain threshold after being expressed in the spinal cord as a consequence of conditional stimulation [40]. The product of c-Fos gene activation is FOS that together with JUN (another protein product of transcription factors) forms the activation protein-1 transcription factor (AP1) [40]. AP1 binds to a DNA binding site coding the preprodynorphin gene [39] that has dynorphin as its main product. Studies confirmed that intrathecally injected dynorphin produced allodynia [42, 43]. Further studies revealed that introduction of NMDA antagonists prevented allodynia concluding that the main contributing factor for the development of allodynia was the effect of dynophilin and its activity on NMDA receptors [40].

Zif268 is a zinc–finger transcription factor and an immediate early gene defined by its response to nerve growth factor treatment leading to neuronal differentiation. It was found to be elevated in the hippocampus as a consequence of tetanic stimulation and spinal cord post-CS [8, 44] hence it might have a role in LTP maintenance [45]. Results of our study determined that the number of Zif268 positive cells increased from the first hour post-CS and has reached its maximal value at 2 h, followed by a gradual decrease by 12 h. The total number of Zif268 positive cells at each time point observed was slightly higher that the number of Arc positive nociceptive cells, but lower than the number of c-Fos positive cells. During their experiments, Rygh et al [8] ablated superficial neurons in the spinal cord containing NK-1 receptors and thereafter were not able to induce facilitation in WDR (Wide Dynamic Range) neurons while Zif268 showed reduced expression in superficial neurons [8]. Intrestingly, Zif268 knockout mice showed impaired formation of late phase LTP in the dentate gyrus [45]. It was therefore proposed that Zif268 could be implicated in the generation of late LTP [45].

As previously mentioned, many studies focused on particular time points (1, 2, 3 h; [21–23]) and specific areas of the spinal cord (L4- L5; L3-L5; L2-L6; [1, 23–25]) leaving results and the methodological approach to CS IEGP alteration in the SC fragmented and incomplete. It was therefore of great scientific importance to conduct a comprehensive study on the temporal and spatial changes in IEGP expression in the SC following CS of the sciatic nerve. Based on ideas from many previous studies from our and other groups, we conducted our experimental research combining and extending the previously established SC boundaries. This work address the need for a reference starting point for further research work in this field, and is up to present day, the only one of its kind conducted on this topic.

## Experimental Procedure

### Animals and surgery

Female Sprague-Dawley rats, 2–3 months of age, weighing 240–300 g were used (NTac:SD, Taconic Europe, Ejby, Denmark). The animals had free access to food and water and were held on a 12/12-h light/dark cycle. The animals were anesthetized with 1.7–2.2 mg/kg urethane (250 mg/ ml in sterile water) injected intra-peritoneal. Animals were then checked for presence of pedal and corneal reflexes whose absence indicated an appropriate level of anesthesia. After shaving of the surgery areas the rats were transferred to a heating pad and during the whole process of stimulation and waiting, the animal body temperature was kept on 37°C.

The left sciatic nerve was dissected from the surrounding muscles on the thigh and a total length of 1–2 cm of the nerve was visible. Proximal to the nerve division, the nerve was placed in a bipolar silver hook electrode (2 mm distance between hooks). The hooks were isolated from the surrounding tissue by means of elastic plastic film (Parafilm, American Can Company, USA).

Eighteen animals were divided in six groups with three animals per group. CS was performed on five groups while the control group underwent all the procedures except receiving CS (sham operated group). Each group of animals was stimulated by using the same CS and then kept anesthetized for different periods of time on individual heating pads before perfusion. The first, second, third, fourth and fifth group was perfused one, two, three, six and twelve hours after stimulation, respectively. The sixth group of rats, the sham operated control group, did not receive CS and was perfused after an idle period of two hours after surgery.

### Sciatic nerve stimulation

All animals except the control group received a conditioning stimulation (CS) consisting of 10 stimulus trains, with stimulus duration of 0.5 ms, amplitude of 7.2 mA, a frequency of 100 Hz, train duration of 2 s and 8 s intervals between trains. This amplitude has previously been shown to be approximately four times the threshold for C-fiber evoked neuronal firing [1]. Stimulation with high frequency bursts of electrical pulses are shown to induce LTP in the dorsal horn of intact animals [18]. CS was given via a PC with Spike2 software coupled via a Digitimer 1401 interface to a stimulator (Neurolog Systems with Stimulus isolator NL800) connected to the bipolar silver hook. After stimulation, the animals were transferred to and kept on heating blankets during the post stimulation period until the time of perfusion.

### Immunohistochemistry

Our goal was to stain for three IEGPs, Arc, c-Fos and Zif268. Therefore we divided all samples (1, 2, 3, 6, 12 h post CS and contol) (N = 3 per time point; total N = 18) in three sets (one set for each IEGP containing all time points and control). Each set of animals consisted of one animal from each group (sham, 1, 2, 3, 6, 12 hours). After a pre-defined post-stimulation period for each animal group the absence of pedal and corneal reflexes were revalidated. If reflexes were found to be present, an additional volume of urethane anesthetic was provided until reflexes disappeared. Animals with abolished reflexes were trans-cardially perfused with 4% paraformaldehyde (PFA) in 0.1 M phosphate buffer, pH 7.4 kept at 4°C. The spinal cord was dissected, post–fixed for 1h in 4% PFA and cryo-protected overnight in a 30% sucrose solution.

The rat’s scull was thereafter fixed in a stereotaxic frame with earplugs and a maxillar clamp. After marking the surgical incisions on the skin, one cranial-caudal incision was made from the occipital region to the top of the inter-ilium bone line. The incision was continued along the line of the vertebral processi spinosi through the subcutaneous fatty tissue to reveal the processi spinosi and the paravertebral musculature [46].

A piece of the spinal cord consisting of the Th13- L5 segments was dissected out. By counting the spinal roots and using the anatomy of the lumbar thickening, the spinal cord was cut at the lumbar thickening between the L2 and L3 segments. The cranial section consisted of Th13-L2 segments while the caudal section consisted of the L3-L5 segments of the spinal cord. These two pieces were separately frozen in custom made aluminum foil wells (Ø 8 mm) in dry ice and kept at -80°C. Both segments of each spinal cord were transverse sectioned in 20 μm thick sections that were mounted onto Superfrost GOLD slides (Braunschweig, Germany). Each slide contained a number of sections for each time point and control sections. In control group underwent surgery including mounting of the silver hook electrodes without actually delivering CS, with a two hour post-surgical idle time period prior to perfusion.

SC sections were first washed in PBST (0.5% Triton X in PBS) and blocked in blocking buffer for 1 h (3% horse serum, 0.5% Triton X in PBS). Afterwards they were incubated overnight in primary antibody diluted in PBST (0.5% Triton X), 3% normal horse serum at 4°C. After three 5 min washes in PBST, the sections were incubated with biotinylated secondary antibody in PBST for 2 h at room temperature. Sections were then washed 3 times 5 min in PBST and incubated in streptavidin- HRP diluted in PBST for 1 h, washed again in the same way, and 3,3'-Diaminobenzidine (DAB) stained for 8 min approximately at room temperature under a microscope to control the color development. The slides were then washed in Milli-Q water three times and stained for 5 min with 0.1% Cresyl Violet (pre heated to 50°C). The slides were then washed 3 times with Milli-Q water and subsequently immersed 3 minutes in each of four baths with increasing ethanol concentration (75%, 90%, 96%, and 96%) and 3 min in two 100% xylene baths. The sections were cover-slipped with DPX mounting medium, and were dried in RT 24 h before imaging.

Mouse anti-Arc monoclonal antibody (1:300 dilution) was purchased from Santa Cruz Biotechnology (cat. #sc-17839), rabbit anti-c-Fos (1:1000 dilution) polyclonal antibody was from Calbiochem (cat. #PC38T) and rabbit anti-Zif 268 (Egr-1) (1:300 dilution) was from Santa Cruz Biotechnology (cat. # sc-110). Secondary antibodies were biotin-conjugated anti-mouse IgG (cat. #PK-4002 VECTASTAIN ABC Kit, Vector laboratories) or biotin-conjugated anti-rabbit IgG (cat. #PK-6101 VECTASTAIN ABC Kit, Vector laboratories).

### Immunofluorescence

Sections for fluorescent staining for co-localization studies were washed in PBST (0.5% Triton X in PBS) and immersed for 1 h in blocking buffer (6% horse serum, 0.5% Triton X in PBS). They were afterwards incubated overnight in primary antibody diluted in PBST, 6% normal horse serum at 4°C. They were afterwards incubated overnight in primary antibody diluted in PBST (0.5% Triton X), 3% normal horse serum at 4°C. After three 5 min washes in PBST, slides were incubated in secondary fluorescein marked antibody for 2 h at RT in dark. After two 45 min washing procedures in PBST, sections were cover slipped with DAPI mounting media (Vector Vectashield mounting medium with DAPI; H-1500) and cover slips (Assistant 50x24 mm, No. 1014). Readymade slides were kept at +4°C for 24 h.

For a 24 h period after cover slipping, slides were kept in dark at 4°C. Imaging was conducted with a Zeiss Axio Imager Z1 upright fluorescence microscope equipped with a mercury Arc lamp (HXP 120), a 40× oil immersion objective (EC Plan-NEO FLUAR 40×/1.3 Oil), single pass fluorescent filters for DAPI (488049–0000), DsRed (1114–101), infrared (488050–0000) and FITC (1114–459) spectra, and a CCD camera (AxioCamMRm). For each experiment, exposure times were carefully chosen to avoid saturation and all images were taken on the same day using the same exposure times. Mouse anti-Arc monoclonal antibody (1:200 dilution Santa Cruz Biotechnology, cat. #sc-17839), rabbit anti-c-fos (1:1000 dilution Calbiochem, cat. #PC38T, Darmstadt, Germany), rabbit anti-Zif 268 (1:200 dilution Santa Cruz Biotechnology, cat. # sc-110), mouse anti-GFAP (GA5) (1:200 dilution, Santa Cruz Biotechnology, cat. # sc- 58766) and rabbit anti- Arc (H300) (1:200 dilution, Santa Cruz Biotechnology, cat. # sc- 5325). Secondary antibodies were donkey anti-mouse Alexa fluor 555 (1:200 dilution, cat. #A-31570; Molecular probes, Invitrogen) and donkey anti-rabbit Alexa fluor 488 (1:200 dilution, cat. # A-31572Molecular probes, Invitrogen). Specificity of staining was determined by eliminating the primary antibody from the staining process. These procedures lead to the elimination of all unspecific staining.

Confocal images were acquired at the Molecular Imaging Center (MIC), Department of biomedicine, University of Bergen, using a Leica SP5 laser scanning microscope (Leica Microsystems, Germany), with a resonant scanner, 40x oil immersion objective and 1.7x or 7-10x electronic zoom for low and high magnifications, respectively. Imaris software (Bitplane) was used for image processing. Images represent maximum projections of 6–10 μm and 4–6 μm thick optical sections for low and high magnifications, respectively.

### Cell quantification

The sections were code marked by our technician before the process of counting without identification of the pretreatment of animals. Cell counting was done manually, and was performed by the first author (O.B.) on DAB stained cells. Cells were defined as positive if they showed a clear staining of the nucleus. The counts were averaged in portions of 4 sections. The stimulated and non-stimulated sides from all dorsal horn sections were imaged (Nikon Eclipse E-600; NIS elements software; resolution 2560x1920; color depth 24-bit RGB) with 100 x magnification. An average of the number of positive cells was calculated for each animal as an average number of 4 sections (400 μm) of spinal cord (n = 3). For co-localization analyzes, the described procedure was done at the level of L3/L4. The average number of all Arc positive cells was compared separately with the number of co-localized cells positive for Arc and c-Fos, and for Arc and Zif268.

### Immunoblot analysis

Animals were divided into three sets consisting of five animals in each set. The first and the second sets of animals received CS as described in the previous experiments and underwent a 2 h or a 3 h idle period after stimulation, respectively. The third set (sham group) underwent all surgical manipulations, did not receive CS and was sacrificed 2 h after surgery. After the pre-defined idle period, animals were sacrificed, and the L3-L5 spinal segments of the spinal cord (of approximately 8mm length) were rapidly removed, put on an ice-cold plate and divided in four quadrants. By one sagittal and one latero-lateral cut, the spinal cords were divided onto one dorsal-stimulated side (DS), dorsal- non-stimulated side (DNS), ventral- stimulated side (VS) and one ventral- non-stimulated side (VNS). After separation all tissue samples were sonicated in radio-immuno-precipitation assay buffer (RIPA).

For immunoblotting we used Arc C7 (1:500; Santa Cruz Biotech, sc-17839) and GAPDH (1:1000; Santa Cruz Biotech, sc-32233). Equal amounts of protein lysate samples were loaded on 8% SDS- PAGE gels and were run 30 min at 60V and 1.5 to 2 h on constant voltage of 100V. Separated proteins were firstly transferred to HyBond ECL nitrocellulose membranes (Amersham, Little Chalfont, UK) at a constant voltage of 100 V for 1.5 h. All membranes were checked for proper protein transfer with Ponceau. After a three times five minute wash cycle in TBST, the membranes were subjected to a blocking procedure with blocking buffer (3% non-fat dry milk, 0.1% Tween and Tris-buffered saline (TBST) for 1 h on a gyro-rocker at room temperature. The primary and the secondary antibodies were diluted in 3% non-fat dry milk in TBST. The membranes were incubated with primary antibody overnight at 4°C with constant shaking. After three washing cycles in TBST, membranes were incubated in horseradish peroxidase (HRP) conjugated secondary antibody dissolved in 3% non-fat dry milk in TBST for 1 hour. The blots were afterwards washed three times with TBST and proteins visualized using enhanced chemo-luminescence (ECL Western Blotting Analysis System, Amersham Pharmacia Biotech, Norway)

### Ethical statement

The experiments were approved by the Norwegian Committee on Research in Animals, and were carried out in accordance with the European Communities Council directive of 24 November 1986 (86/609/EEC). Efforts were made to minimize the number of animals used, and experiments were designed to minimize suffering.

### Statistics

Statistics were done with the Graphpad Prism 6.0 statistical package. Statistical significance was accepted at the 5% level. For statistical evaluation of cell numbers, the values for each animal were calculated as the average numbers of cells per section over lengths of spinal cord of 400 μm (four 20 μm sections with a 80 μm distance between each section).

Cell numbers are shown graphically as means± S.E.M. (N = 3) for each 400 μm of lumbar spinal cord studied. Graphical presentations were made by Sigma Plot 9.0 software.

For all time points (1, 2, 3, 6, 12 h), the comparisons between the post-stimulation groups and the control groups (for all IEGPs) were done by means of two-way analysis of variance (ANOVA). During ANOVA, stimulation and spinal location were used as independent variables while the number of cells was used as the dependent variable. If a significant effect of stimulation was found, that set of animals was compared with the sham group with post hoc Sidak’s tests.

For fluorescent co-localization, statistical analysis of significance between the control side (non-stimulated) and stimulated side was done by means of Student’s t-tests. For statistical analysis, the percentages of co-localized cells of the number of Arc-stained cells were calculated for both analyzed groups (Arc+C-Fos; Arc+Egr-1). The number of co-localized cells were counted in four random view fields (63x) per every 100 μm of 400 μm sections (dorsal horn) per animal (N = 3; 2 h post CS).

For confocal analysis, the number of NeuN, Dapi and Arc positive cells were manually counted for each time point, for all animals (N = 3 per time point), in three random view fields (40 x) of all sections (3 sections per animal) per every 100 μm at -2 mm ± 100 μm (dorsal horn). Then the average percentage of Arc/NeuN co-localization was calculated for each time point together with standard error of the mean (SEM).

Analysis of the Western blot results was done by performing unpaired Student’s t-tests on means of normalized densities of stimulated, non-stimulated and control tissue samples (N = 5 in each group).
